# Students’ perception of educational environment at Aga Khan University Medical College, Karachi, Pakistan

**DOI:** 10.12669/pjms.323.9562

**Published:** 2016

**Authors:** Rehana Rehman, Kulsoom Ghias, Syeda Sadia Fatima, Mehwish Hussain, Faiza Alam

**Affiliations:** 1 Dr. Rehana Rehman, PhD. Department of Biological and Biomedical Sciences, Aga Khan University, Stadium Road, Karachi, 74800, Pakistan; 2 Dr. KulsoomGhias, PhD. Department of Biological and Biomedical Sciences, Aga Khan University, Stadium Road, Karachi, 74800, Pakistan; 3 Dr. Syeda Sadia Fatima, MPhil. Department of Biological and Biomedical Sciences, Aga Khan University, Stadium Road, Karachi, 74800, Pakistan; 4 Dr. Mehwish Hussain, PhD. Department of Research, Dow University of Health Sciences, Karachi, Pakistan; 5 Dr. Faiza Alam, MPhil. Department of Biological and Biomedical Sciences, Aga Khan University, Stadium Road, Karachi, 74800, Pakistan

**Keywords:** Dundee ready education environment measure (DREEM), Learning environment, Medical students

## Abstract

**Objective:**

To assess educational environment in Aga Khan University Medical College (AKUMC) by Dundee Ready Educational Environmental Measure (DREEM) scale.

**Methods:**

A cross-sectional survey of students at the AKUMC with simple random sampling was carried out from June 2014 till March 2015. Responses in five subscales were used to calculate DREEM scores.

**Results:**

The average DREEM score was 125.77±16.8 with a reliability of 91.3%. With regards to subscales, on the 12-item students’ perceptions of learning (PoL) subscale, the maximum score was 48; 11 items of students’ perceptions of teachers (PoT) had a maximum score of 44; students’ academic self-perceptions (ASP) identified by 8 items showed maximum score of 32; students’ perceptions of atmosphere (PoA) with 12 items obtained maximum score 48 and students’ social self-perceptions (SSP) subscale of 7 items had a maximum score of 28.

**Conclusion:**

Students perceived a positive learning environment at AKUMC Karachi.

## INTRODUCTION

Learning environment is one of main factors for regulating student learning and for evaluation of medical education programs.^1^ The effects of educational environment (EE) are important determinants of medical students’ attitudes, knowledge, skills, progression, behaviors as well as academic achievements.^2^ Understanding of an institutional EE allows for effective curricular management and informs practice that may particularly affect students coming from diverse educational and cultural backgrounds.

The Aga Khan University Medical College (AKUMC) was the first private medical college established in 1983 in Pakistan. An integrated, patient-orientated, clinical presentations-based, spiral curriculum, which relies on multimodal pedagogical approaches, including problem-based learning (PBL) was introduced in 2002. Besides PBL, other strategies including interactive lectures, laboratory sessions, tutorials, clinical skills sessions and field visits are implemented to stimulate active learning. Though the programme has undergone several internal and external reviews since its inception, the EE and students’ satisfaction has not been explicitly evaluated.

A number of instruments are available for estimation of EE of undergraduate medical students, of which the Dundee Ready Education Environment Measure(DREEM) was designed to accurately measure the EE in medical institutes.^3^ It is based on a five point Likert scale measuring five dimensions of the environment^4^,^5^ and considered a valid and reliable tool, globally accepted for measuring the EE and is valuable for highlighting areas of concern identified by medical students, including educational climate, academic achievement, and social support.

The objective of this study was to assess the educational environment prevalent in a private medical college in Pakistan (AKUMC) using DREEM scale and subscales.

## METHODS

### Description of Instrument (DREEM)

DREEM is a survey tool developed to quantitatively measure students’ perceptions of EE within a health profession educational setting. The DREEM survey consists of 50 items or statements, each scored 0–4 on a 5-point Likert Scale (0 = strongly disagree (SD) to 4 = strongly agree (SA)). There are nine negatively stated items: which are scored in reverse (0 = strongly agree to 4 = strongly disagree). Mean scores for individual items are calculated with a maximum score of 4.0 for each item. The 50 items are subsequently analyzed within five subscales created by combining specific items; 1) students’ perceptions of learning (PoL) (12 items, maximum score 48), 2) students’ perceptions of teachers (PoT) (11 items, maximum score 44), 3) students’ academic self-perceptions) (8 items: maximum score 32), 4) students’ perceptions of atmosphere (PoA) (12 items, maximum score 48) and 5) students’ social self-perceptions (SSP) (7 items: maximum score 28). After entry and compilation of data scale, scores and subscale scores were calculated. Subscale PoL was constructed by adding responses of items 1, 7, 13, 16, 20, 22, 24, 25, 38, 44 and 47. Responses of items 2, 6, 8, 9, 18, 29, 32, 37, 39, 40 and 50 were added to create the subscale PoT. Items 5, 10, 21, 26, 27, 31, 41 and 45 were summed up for creating the subscale ASP and PoA was constructed by adding responses of items 11, 12, 17, 23, 30, 33, 34, 35, 36, 42, 43 and 49. The subscale SSP was created by summing the responses of items 3, 4, 14, 15, 19, 28 and 46. The overall DREEM score was computed while adding scores of all subscales.

### Statistical Analysis

The socio-demographic characteristics of students are presented in terms of frequency and percentages. To describe statistics of responses of each items and scores, mean with standard deviation and standard error of mean (SEM) were computed. Cronbach’s alpha was computed to measure consistency within the responses of students along with item total correlation to check correlation of each item with total score. Prior to performing each inferential analysis, the normality assumption of scores were assessed via Shapiro-Wilk’s test and found non-normal. Therefore, non-parametric analyses were performed to proceed for comparative and association analyses. To determine the relationship between scales scores, Spearman’s correlation statistic was computed. The threshold value for indicating significance of findings was set at 0.05 level.

## RESULTS

### Participants Characteristics

Of the 416 participants, 235 were female. Most of the participants came from a British education background i.e. GCE (O-level and A-level). Half of the students lived in hostel. The participation from first year students was 24% (n = 100), second year 21.6% (n = 90), third year 22.1% (n = 92), fourth year 21.9% (n = 91) and fifth year 10.3% (n = 43).

### Item Descriptive Statistics

We determined an item that scored more than 2.5 as good and items that scored more than or equal to 3 as an excellent measure. The item “I have good friends in this school” received highest average score (3.18±0.79) followed by “Much of what I have learnt seems relevant to a career in healthcare (3.05±0.75). These were also the only items received score of more than 3.0. Two items that received an average score of 3 were “The teachers are knowledgeable” and “My social life is good”. Other than these 4 items, 27 items had an average score of more than 2.5. There were 3 items with an average score of less than 2.0 indicating consideration should be made to improve these matters. These items were “The teaching over-emphasizes factual learning” (1.65±1.01), “The students irritate the teachers” (1.67±1.17) and “I am able to memorize all I need” (1.83±1.08).

### Scales Description

The descriptive statistics of each subscale in the questionnaire are presented in Table-I. The average overall DREEM scale score by the students in this study was 125.7±16.8. The reliability of the scale was 91.3%, indicating excellent consistency of responses by students. The reliability is be marginally improved if any of the following three items are deleted: “The teaching over-emphasizes factual learning”, “The student irritates the teacher” and “Cheating is a problem in this school”. The subscales students’ PoA and students’ PoL showed excellent reliability and are interpreted as a more positive approach corresponding to the mean values. Students’ PoT also showed good consistency in the responses with are liability of 73.1%. The values of Cronbach’s alpha for students’ ASP and students’ SSP were 68% and 62%,

**Table-I:** Descriptive Statistics of Scores.

	*Mean*	*SD*	*Subscale score interpretation*	*Alpha*
Perception of Learning (PoL)	29.73	6.480	A more positive approach	0.802^!^
Perception of Teachers (PoT)	27.13	5.630	Moving in the right direction	0.731^#$^
Academic Self-Perception (ASP)	20.93	4.011	Feeling more on the positive side	0.680^^&^
Perception of Atmosphere (PoA)	31.70	6.928	A more positive atmosphere	0.828*
Social Self-Perception (SSP)	17.63	15.276	Not too bad	0.62
Total DREEM Score	125.68	18.798	More positive than negative	0.913^+%^

! Improves to 0.853 if The teaching over-emphasizes factual learning is deleted

# Improves to 0.721 if The teachers ridicule the students is deleted

$ Improves to 0.75 if The students irritate the teachers is deleted

^ Improves to 0.683 if I am able to memorize all I need is deleted

& Improves to 0.696 if Learning Strategies which worked for me before continue to work for me now is deleted

* Improves to 0.837 if Cheating is a problem in this school is deleted

+ Improves to 0.917 if The teaching over-emphasizes factual learning is deleted

% Improves to 0.915 if The students irritate the teachers and Cheating is a problem in this school are deleted

Correlation analysis revealed that atmosphere and learning environment was highly positively associated with overall educational environment (r = 0.856 and 0.808 respectively). ASP played the least, but significant role in this regard (r = 0.694). Among the subscales, the highest correlation was observed between perception towards teaching and atmosphere (r = 0.649) (Table-II).

**Table-II:** Correlation of subscales and overall scale.

	*PoL*	*PoT*	*ASP*	*PoA*	*SSP*
PoT	.500**				
ASP	.514**	.370**			
PoA	.560**	.649**	.498**		
SSP	.468**	.383**	.514**	.575**	
Overall DREEM	.808**	.755**	.694**	.856**	.712**

** Correlation is significant at the 0.01 level (2-tailed).

PoL: Perception of Learning.

PoT: Perception of Teachers.

ASP: Academic Self-Perception.

PoA: Perception of Atmosphere.

SSP: Social Self-Perception.

## DISCUSSION

Assessment of the climate of an educational program is a valuable tool to ensure quality and to identify areas of improvement. The educational environment is not just a measure of student satisfaction, but also affects student behavior and predicts achievement.^6^-^8^ EE can best be interpretedin terms of overall and subscale scores, as well as item analysis of responses acquired by DREEM.^9^

The mean total score for the DREEM in this study was 126, which is greater than or comparable to other studies from medical colleges reported in the literature from around the world.^10^-^21^ The score classifies the undergraduate medical education program at Aga Khan University, Karachi as more positive than negative as per the interpretation suggested in the literature.^22^

Subscale analysis shows the most positive responses in the domains of Students’ PoA and PoL (Table-I). The total DREEM scores of these subscales can be interpreted as a more positive approach. Other studies that have used DREEM to assess EE in Pakistani medical colleges reported the highest scores in the domains related to Students’ Self-perceptions and lowest scores in the PoL and PoT Perception subscales.^9^,^10^ In this study, highest reliability was in PoL and PoA, which is in contrary to other studies that reported highest scores in the domains related to SSP and lowest scores in the PoL and PoT subscales.^10^

In the PoL subscale, the most positive responses received were in relation to being given clear learning objectives, teaching helping develop competence/learning capabilities and well-focused teaching with encouragement to participate in class and towards active learning. Mean scores in the PoT subscale show that students agree that the teacher are knowledgeable, well-prepared for their classes and patient with their audience. However, there is room for improvement in providing constructive criticism and feedback, which was identified in other DREEM studies as well^19^,^23^ and is a critical role of a teacher.^24^ The faculty members receive training and the curriculum at AKUMC has opportunities built-in for continuous feedback, which can be reinforced.

The ASP subscale reveals that students feel they are being well-prepared for the medical profession and are learning what is relevant to a career in healthcare, including empathy and problem-solving. The undergraduate medical program follows a problem-based hybrid curriculum and offers opportunity to develop problem-solving and communication skills. The lowest scoring item in this subscale was related to the students’ perceived ability to memorize all that is needed. In light of this and the response to the item related to over-emphasis on factual learning in the SPL subscale, there appears to be a need to review teaching, learning and assessment strategies and focus on application of knowledge rather than memorization.

The responses to items in the PoA subscale indicate a positive learner-centered, relaxed atmosphere as suggested by related items in other subscales. However, timetabling of curricular activities should be re-visited to ensure that teaching time is being used appropriately, as this was one of the lower scoring items. While some level of stress is expected and is present as reflected in the item addressing enjoyment of course being outweighed by stress, the ability to manage stress and develop appropriate coping mechanisms is important. The institution needs to play a proactive role in providing adequate resources to support students mentally and academically throughout the course. The sports and recreational facilities available at AKUMC are often appreciated by students in the context of preventing burn out.

Response to the item on students’ perception of a good support system for students who experience stress in the SSP subscale suggests that this area needs further strengthening. This is already underway through an integrated system providing mentorship, academic and mental health support and access to counseling. It is critical that students are made aware of the availability of these services. The students’ perception of cheating practices is a matter of concern. While the mean score suggests that students are mostly neutral about cheating being a problem at the school, another study within the same program has previously investigated students’ attitudes and behaviors towards plagiarism, lying, cheating and stealing and shown that the ability to identify acts of academic misconduct does not deter students from engaging in the behavior themselves.^25^ Professionalism is of paramount importance in medical graduates and one way to inculcate it is through good role-modeling and discouragement of unethical and immoral practices, such as cheating. While the institution has a zero tolerance with regards to cheating, it is critical to explore why cheating might take place and instead of only punitive actions, address the issue proactively by giving attention to the hidden curriculum, teaching and role-modeling ethics and professionalism and creating a culture of integrity.^19^,^26^ The SSP scale was one of the highest performing in the entire survey, with responses indicating that they make good friends in the school and have a good social life.

### Strengths and limitations

This study had an excellent response rate and reliability. A limitation of the study was lack of open ended questions or any focused group discussion (FGD). Generalization is also not possible since the data was collected from one institution.

## CONCLUSIONS

The overall educational environment at the Aga Khan University Medical College in Karachi, Pakistan is more positive than negative, which is comparable to other programs studied using the DREEM inventory in the country, region and beyond. The results of this study provide an insight into the program strengths, such as institutional atmosphere and areas of improvement, such as peer and faculty support. These scores can be used to plan future strategic decisions. In the future, undergraduate clinical education environment can also be measured.
